# A novel long intergenic non-coding RNA, Nostrill, regulates *iNOS* gene transcription and neurotoxicity in microglia

**DOI:** 10.1186/s12974-020-02051-5

**Published:** 2021-01-06

**Authors:** Nicholas W. Mathy, Olivia Burleigh, Andrew Kochvar, Erin R. Whiteford, Matthew Behrens, Patrick Marta, Cong Tian, Ai-Yu Gong, Kristen M. Drescher, Peter S. Steyger, Xian-Ming Chen, Annemarie Shibata

**Affiliations:** 1 School of Medicine, Creighton University, 2500 California Plaza, Omaha, NE 68178-0010 USA; 2 Biology Department, Creighton University, 2500 California Plaza, Omaha, NE 68178-0100 USA; 3 University of Nebraska College of Medicine, 987020 Nebraska Medical Center, Omaha, NE 68198-7020 USA

**Keywords:** Microglia, Long non-coding RNA, lncRNA, Nostrill, NR_126553, 2500002B13Rik, NF-κB, iNOS, NO, Inflammation, Neurotoxicity

## Abstract

**Background:**

Microglia are resident immunocompetent and phagocytic cells in the CNS. Pro-inflammatory microglia, stimulated by microbial signals such as bacterial lipopolysaccharide (LPS), viral RNAs, or inflammatory cytokines, are neurotoxic and associated with pathogenesis of several neurodegenerative diseases. Long non-coding RNAs (lncRNA) are emerging as important tissue-specific regulatory molecules directing cell differentiation and functional states and may help direct proinflammatory responses of microglia. Characterization of lncRNAs upregulated in proinflammatory microglia, such as NR_126553 or 2500002B13Rik, now termed *Nostrill* (iNOS Transcriptional Regulatory Intergenic LncRNA Locus) increases our understanding of molecular mechanisms in CNS innate immunity.

**Methods:**

Microglial gene expression array analyses and qRT-PCR were used to identify a novel long intergenic non-coding RNA, Nostrill, upregulated in LPS-stimulated microglial cell lines, LPS-stimulated primary microglia, and LPS-injected mouse cortical tissue. Silencing and overexpression studies, RNA immunoprecipitation, chromatin immunoprecipitation, chromatin isolation by RNA purification assays, and qRT-PCR were used to study the function of this long non-coding RNA in microglia. In vitro assays were used to examine the effects of silencing the novel long non-coding RNA in LPS-stimulated microglia on neurotoxicity.

**Results:**

We report here characterization of intergenic lncRNA, NR_126553, or 2500002B13Rik now termed Nostrill (iNOS Transcriptional Regulatory Intergenic LncRNA Locus). Nostrill is induced by LPS stimulation in BV2 cells, primary murine microglia, and in cortical tissue of LPS-injected mice. Induction of Nostrill is NF-κB dependent and silencing of Nostrill decreased inducible nitric oxide synthase (iNOS) expression and nitric oxide (NO) production in BV2 and primary microglial cells. Overexpression of Nostrill increased iNOS expression and NO production. RNA immunoprecipitation assays demonstrated that Nostrill is physically associated with NF-κB subunit p65 following LPS stimulation. Silencing of Nostrill significantly reduced NF-κB p65 and RNA polymerase II recruitment to the iNOS promoter and decreased H3K4me3 activating histone modifications at *iNOS* gene loci. In vitro studies demonstrated that silencing of Nostrill in microglia reduced LPS-stimulated microglial neurotoxicity.

**Conclusions:**

Our data indicate a new regulatory role of the NF-κB-induced Nostrill and suggest that Nostrill acts as a co-activator of transcription of iNOS resulting in the production of nitric oxide by microglia through modulation of epigenetic chromatin remodeling. Nostrill may be a target for reducing the neurotoxicity associated with iNOS-mediated inflammatory processes in microglia during neurodegeneration.

**Supplementary Information:**

The online version contains supplementary material available at 10.1186/s12974-020-02051-5.

## Background

Systemic inflammation due to pathogenic infection is a direct cause of dysregulated neuroimmune responses and is linked to several neurodegenerative pathologies of the central nervous system (CNS) [1–7]. Microglia, the principal neuroimmune cells, participate in the immune processes of pathogen clearance contributing to both neurorecovery and neurotoxicity [5]. Microglia exhibit functional plasticity and are able to act as homeostatic surveillance cells [8–11], anti-inflammatory and neuroprotective cells [5, 7, 12–15], or pro-inflammatory and neurotoxic cells [1, 16, 17]. Microglia express a diverse transcriptome indicative of their complex functional roles in the CNS [18, 19]. Microglia continually and rapidly respond to changes in the CNS environment [20–22]. This functional flexibility requires that microglia regulate the timing and rate of gene transcription [18, 21]. Upregulation of microglial proinflammatory states caused by the transcription of specific genes that underlie neuroinflammatory processes is likely to contribute to neurotoxicity.

Long non-coding RNA (lncRNA) are RNA transcripts that are longer than 200 nucleotides, are frequently polyadenylated, and do not contain open reading frames. LncRNAs can be classified into several subtypes *antisense*, *intergenic*, *overlapping*, *intronic*, *bidirectional*, and are processed according to the position and direction of transcription in relation to other genes. They are emerging as important tissue-specific regulatory molecules directing proper cell differentiation and development [14, 23–26]. Intergenic are the largest subclass of lncRNAs, and are referred to as long intergenic non-coding RNAs (lincRNAs) [23, 25, 27]. LincRNA expression is cell and tissue type specific [24] and thousands of lincRNAs have been identified in the mouse genome. LincRNAs are prime candidates for regulating microglial polarity because many lincRNAs are early primary response genes whose expression is stimulated by environmental signals [25, 28]. LincRNAs are associated with human inflammatory disease and neuropathologies [27, 29, 30]. LincRNAs can function in cis, recruiting protein complexes to their site of transcription and thus creating a locus-specific address. They can also function in trans to regulate distantly located genes. Many identified lincRNAs function in the nucleus to guide chromatin modifiers such as H3K9 methyltransferase and polycomb repressive complex to specific genomic loci to repress gene transcription. In previously published collaborative work, we have identified that TLR4-stimulated macrophages and microglia upregulate lincRNA-Cox2. Upon bacterial lipopolysaccharide (LPS) stimulation of TLR4, lincRNA-Cox2 interacts with the nucleosome remodeling complex SWI/SNF to modulate pro-inflammatory NF-κB signaling. SWI/SNF-associated histone acetylation causes transactivation of late-primary inflammatory response genes in LPS-stimulated microglia [31]. We have also shown in macrophages and microglia that lincRNA-Tnfaip3 transcript interacts with components of the Hmgb1 complex, and an NF-κB/Hmgb1/lincRNA-Tnfaip3 complex assembles in microglial cells in response to LPS stimulation [32]. These preliminary data provide novel and exciting evidence that lincRNAs may be involved in microglia plasticity and polarization in response to environmental cues. Therefore, lincRNAs may participate in pathogenesis of various inflammatory and neurodegenerative diseases making them targets for therapeutic interventions.

Pro-inflammatory microglia, stimulated by environmental microbial signals such as bacterial LPS are neurotoxic and are associated with pathogenesis of neurodegenerative disease [33, 34]. Pro-inflammatory microglia enhance phagocytosis and secrete inflammatory cytokines, chemokines, arachidonic acid, reactive oxygen and nitrogen species, and growth-inhibiting proteins [4, 6]. Recent network analyses provide evidence for the upregulation of several lncRNAs, including lincRNAs, in response to LPS stimulation in microglial cell lines [32, 35]. Additionally, lincRNAs are associated with the pathogenesis of several inflammatory diseases and neurological disorders [26, 32, 36–38]. In this study, we report that the previously uncharacterized lincRNA (NR_126553) that we termed *Nostrill* (iNOS Transcriptional Regulatory Intergenic LncRNA Locus) is induced by inflammatory mediators and controlled by NF-κB signaling in microglial cell lines and primary microglia following TLR3, and more dramatically, TLR4 stimulation. Silencing or overexpression of Nostrill in microglial cells influenced iNOS mRNA levels and nitric oxide production. Nostrill is physically associated with NF-κB p65 following LPS stimulation. Knockdown of Nostrill decreased NF-κB p65 and RNA polymerase II recruitment to *iNOS* promoter region and decreased H3K4me3 activating histone modifications. Importantly, blocking the expression of Nostrill in microglia reduced proinflammatory toxicity to primary cultured cortical neurons in cellular assays. Identifying pro-inflammatory lincRNAs such as Nostrill that when silenced reduce microglial neurotoxicity may be useful in developing targeted therapeutic strategies that reduce the neurotoxicity associated with immune responses to pathogenic signals and thereby limit neurodegeneration.

## Methods

### Animals

Animals were housed in AAALAC-accredited facilities, and all experiments were conducted under protocols approved by the Creighton University Institutional Animal Care and Use Committee. C57BL/6 J mice were obtained from The Jackson Laboratory. Mice were housed and bred in the animal care facility at Creighton University under a 12/12 h light/dark cycle with ad libitum access to food. For primary microglial and cortical neuronal cell isolation, animals were treated in strict accordance to the approved Institutional Animal Care and Use protocol #0793. For LPS injection In Vivo Model, animals were treated in strict accordance to approved by the Institutional Animal Care and Use Protocol #1086.

### LPS injection in vivo model

Male and female C57BL/6 J mice of age 6-weeks old were divided into two groups: a vehicle control group receiving intravenous (IV) tail vein injection of Dulbecco’s phosphate-buffered saline (50 μl/10 g, DPBS, Thermo Fisher Scientific, Waltham, MA) or an experimental group receiving an IV injection of LPS at 1 mg/kg (*Escherichia coli* O111:B4, Sigma-Aldrich, St. Louis, MO, USA) in DPBS. At 24 h, mice were anesthetized with ketamine/xylazine and transcardially perfused with cold phosphate-buffered saline (PBS). Mice were weighed before LPS injection and 24 h after injection. LPS injected mice usually lose ~ 10% of the body weight, which can be used as an indication of successful tail vein delivery of LPS. Brain tissue was dissected and immersed in Invitrogen RNALater™ (Thermo Fisher Scientific, Waltham, MA) overnight at 4 °C and stored at −80 °C for Invitrogen TRI Reagent™ (Thermo Fisher Scientific, Waltham, MA) RNA extraction. RT-PCR was performed as described below.

### Microglial cell line culture

BV2 mouse microglia were purchased from American Type Culture Collection (ATCC CRL-2467; Manassas, VA). BV2 cells were maintained in DMEM (Hyclone, ThermoFisher Scientific, Waltham, MA) supplemented with 10% fetal bovine serum (FBS, Hyclone #SH30072.03, Lot No. AXB30110, Thermo Fischer Scientific, Waltham, MA), 1% l-glutamine, 1% penicillin/streptomycin (ThermoFisher Scientific, Waltham, MA). Cells were grown in 100-mm tissue culture dishes at 37 °C in 5% CO_2_ and allowed to reach 80% confluency before passage.

### Primary cortical microglial cell culture

Primary microglial cells were isolated from P0-P2 C57BL/6 J mice (The Jackson Laboratory, Bar Harbor, ME)*.* Use of animals was performed in strict accordance with the Institutional Animal Care and Use committee guidelines as approved by the IACUC committee at Creighton University (protocol #0793). P0-P2 mouse brains were dissected, meninges were removed, and cortices were isolated in ice cold, sterile Ca^2+^/Mg^2+^-free Hank’s balanced salt solution (HBSS, #14025092, Thermo Fischer Scientific, Waltham, MA). Cortices were minced and mechanically dissociated in Ca^2+^/Mg^2+^-free HBSS, with 0.035% sodium bicarbonate (#25080094, Thermo Fischer Scientific, Waltham, MA) and 1 mM pyruvate (pH 7.4, #11360070, Thermo Fischer Scientific, Waltham, MA) following 15 min digestion with 0.25% trypsin-EDTA (#15090046, Thermo Fischer Scientific, Waltham, MA). Trypsin was neutralized with Dulbecco’s Modified Eagles Media (DMEM, Hyclone, Thermofisher Scientific, Waltham, MA) supplemented with 10% FBS (Hyclone #SH30072.03, Lot No. AXB30110, Thermo Fischer Scientific, Waltham, MA) and cells were mechanically triturated. Cell suspensions were strained through a sterile 70-μm nylon mesh strainer and plated onto poly-D-lysine coated 75mm^2^ tissue culture flasks in DMEM plus 10% FBS and 1% penicillin/streptomycin and allowed to reach confluency over 14 days at 37 °C in 5% CO_2_. After reaching confluency, cells were shaken vigorously on an orbital shaker at 220 rpm to remove microglia. Microglial were collected and re-seeded at 0.5 × 10^6^ cells/ml onto tissue culture plates. After 1 h attachment, floating cells were removed and adherent cells were cultured in DMEM plus 10% FBS and 1% penicillin/streptomycin at 37 °C in 5% CO_2_, unless rinsed and switched into Neurobasal media for experiments. Microglial purity was determined using immunocytochemical analysis of cortical cell protein expression (described below).

### Isolation of cortical neurons

Primary cortical cells were isolated from P0-P2 C57BL/6 J mice (The Jackson Laboratory, Bar Harbor, ME) following methods modified from Ahlemeyer et al. [39]. Use of animals was performed in strict accordance with the Institutional Animal Care and Use committee guidelines as approved at Creighton University (Protocol #0793). Briefly, P0-P2 mouse cortices were isolated, minced, and mechanically dissociated as described for primary microglial cell culture. Cortical cell suspensions were washed three times and resuspended with Neurobasal media supplemented with B-27™ Plus Supplement (GibcoBRL #A35828-01, Thermo Fischer Scientific, Waltham, MA) and penicillin/streptomycin (#10378016, Thermofisher Scientific, Waltham, MA) and dissociated with mechanical trituration. Cell suspensions were centrifuged for 5 min at 1000 rpm, resuspended in supplemented serum-free Neurobasal media and plated onto poly-D-lysine (#P0899, Sigma, St. Louis, MO) coated tissue culture plates at density of 1.5 × 10^6^ cells/well in 6-well plates and 5 × 10^5^ cells/well in 24-well plates at 37 °C in 5% CO_2_ for at least 1 week. Each cortical culture was considered a biological replicate and all experiments were performed in triplicate.

### Neuronal—microglial co-cultures

BV2 microglia and neuronal cells were cultured as described above. BV2 microglia were pre-seeded at 4 × 10^4^ cells/well directly onto either 6-well or 24-well, 0.4 μm permeable Transwells® (Corning,Tewksbury, MA) and cultured for 24 h in neurobasal serum-free B27-free media before being suspended above cortical neurons in the co-culture model system (Fig. 7a, created with BioRender.com). BV2 microglia were placed in suspension above cortical neurons and co-cultured for an additional 3 days in unsupplemented Neurobasal media at 37 °C in 5% CO_2_. After co-culture, Transwells® with microglia were removed and cortical neuronal cultures were fixed in culture media plus 3.7% formaldehyde at 37 °C in 5% CO_2_. Cortical neuronal cultures were assessed using immunocytochemistry (described below).

### Immunocytochemistry

Cortical primary microglial or cortical neuronal cultures were fixed with 3.7% formaldehyde in cell culture media, rinsed in PBS. Cells were permeabilized with 0.2% Triton X-100 in PBS for 10 min, washed, and blocked for 1 h in PBS, 0.2% BSA, and 0.2% Triton X-100. Primary antibodies were applied and incubated overnight at 4 °C in PBS, 0.2% BSA, 0.2% Triton X-100. Cells were incubated with anti-Iba-1 (1:200, Abcam Cat #ab178846, RRID:AB_2636859), rabbit anti-GFAP (1:400, Millipore Cat # AB5541, RRID:AB_177521), and mouse anti-beta tubulin III/TUJI (1:200, Millipore Cat # MAB1637, RRID:AB_2210524). Secondary antibodies were applied for 1 h at a concentration of 1:500 for goat anti-rabbit IgG (H + L) Alexa Fluor 488 conjugate and goat anti-mouse IgG (H + L) Alexa Fluor 594 conjugate (Pierce, Rockford, IL). Nuclei were visualized using a DAPI stain (300 mmol, MP Biomedicals, Santa Ana, CA). Qualitative and quantitative analysis of immunocytochemistry was performed by acquiring images with a Leica DMI4000B inverted microscope with a cooled CCD camera (Q Imaging, Surrey, BC) and fluorescent capabilities. The percent of cells expressing cell-specific proteins was determined by counting the number of immunopositive cells for each marker and dividing that number by the total number of cells counted in the field. Quantification of relative fluorescence intensity of protein expression in cortical cell cultures was determined by measuring integrated pixel intensity and mean gray value for the imaged area. For data analyses, 3 fields of the same area with at least 100 cells in each area from 3 separate experiments were analyzed for each condition. In all experiments, images were analyzed with Volocity (PerkinElmer, USA) and ImageQuant (GE Healthcare, USA) software.

### Measurement of cell viability—propidium iodide incorporation

Cell viability was measured using propidium iodide incorporation methods as described by the manufacturer (Invitrogen, #P1304MP, Thermo Fischer Scientific, Waltham, MA). Propidium iodide will permeate dead cells and is used to detect cell death/viability. Briefly, following co-culture with microglia cortical neuronal cultures were RNase-Treated by equilibrating for 5 min in 2X SCC buffer (0.3 M NaCl, 0.03 M sodium citrate, pH 7.0) and then incubated in 100 μg/ml RNase-free RNase in 2X SCC for 20 min at 37 °C. Cells were rinsed three times in 2X SCC and counterstained with 500 nM PI in 2X SCC for 5 min. Cells were rinsed three times in 2X SCC, excess buffer was removed, placed in 1X PBS, and imaged immediately. Neuronal cultures were viewed for propidium iodide (PI) red-fluorescent nuclear and chromosome counterstaining using 1 μg/ml for 5 min using Hoeschst 33342 solution (#62249, ThermoFischer Scientific, Waltham, MA). Images were acquired via the EVOS M5000 cell imaging system (Excitation 535 nm/Emission 617 nm for PI and UV Excitation/Emission460nm for Hoeschst) and images saved for later analysis using Firmware, EVOS FLoid Software (Thermo Fischer Scientific, Waltham, MA). In all experiments, acquired images were analyzed with Volocity (PerkinElmer,USA) and ImageQuant (GE Healthcare, USA) software were used for image analysis and presentation. Experiments were performed in triplicate.

### Small interfering RNAs and transfection

For gene silencing, the small interfering RNA (siRNA) duplexes for mouse Nostrill were synthesized using Integrated DNA Technologies. The siRNA sequences targeting Nostrill were as follows: sense, 5′- CGAGAUAGGCUGAGGACUU -3′; antisense, 5′- AAGUCCUCAGCCUAUCUCG -3′. The nonspecific scrambled siRNA sequence UUCUCCGAACGUGUCACGUUU was used for the control. Cells were treated with siRNAs (final concentration, 60 nM) using Lipofectamine RNAiMAX (Invitrogen, Carlsbad, CA) according to the manufacturer’s instructions. For Nostrill overexpression, Nostrill cDNA was amplified through PCR, inserted into the PTarget (Promega, Madison, WI) expression vector to generate PTarget-Nostrill, and subsequently sequenced. According to the manufacturer’s protocol, cells were transfected with plasmid DNA using Lipofectamine 2000. Quantitative RT-PCR was used to determine the significant alteration of each target gene.

### RT-PCR analysis

For real-time PCR analysis of cytokines, total RNA was isolated from cells with Trizol reagent (Applied Biosystems). An amount of 200 ng total RNA was reverse-transcribed using the iScript Reverse Transcription Supermix (Bio-Rad, Hercules, CA). Comparative real-time PCR was performed using the Invitrogen™ SYBR GreenER™ qPCR SuperMix Universal (Thermo Fisher Scientific, Waltham, MA) on the Bio-Rad CFX96 Touch™ Real-Time PCR Detection System. The sequences for all the primers are listed in Supplementary Table 1. Normalization was performed using Gapdh. Relative expression was calculated using the comparative Ct (ΔΔCt) method.

### Griess analysis

Media collected from microglial cultures were evaluated using a Nitric Oxide Assay Kit to determine nitric oxide composition through measurement of nitrate (NO_3_) and nitrite (NO_2_) levels according to manufacturer’s instructions (#EMSNO, Thermo Fischer Scientific, Waltham, MA). Briefly, 1X reagent diluent, NADH, and nitrate reductase were prepared as recommended in the kit instructions. Samples were diluted 1:2 with 1X reagent diluent and filtered through a 10,000 MWCO filter. NADPH was oxidized with 10 μL of lactate dehydrogenase (1500 U/ml in 30 mM sodium pyruvate) after incubation with nitrate reductase and incubated at 37 °C for 10 min. Nitrate standards were prepared by serial dilution following manufacturer’s instructions. Griess reagents I and II were added to standard, control, and sample wells. Plates were tapped to mix and incubated at room temperature for 10 min. Plates were read using an optical density at 540 ± 20 nm on Synergy HTX multi-mode reader (BioTek US, Winooski, VT). Technical triplicate readings were averaged and experiments were run in biological triplicates.

### RNA immunoprecipitation assay

The formaldehyde crosslinking RIP was performed as described [31]. Briefly, lysates were precleaned with 20 μl of PBS washed Magna ChIP Protein A + G Magnetic Beads (Millipore, Massachusetts). The precleaned lysate (250 μl) was then diluted with the whole cell extract buffer (250 μl), mixed with the specific antibody-coated beads, and incubated with rotation at 4 °C for 4 h, followed by 4 times washing with the whole cell extract buffer containing protease and RNase inhibitors. The collected immunoprecipitated RNP complexes and input were digested in RNA PK Buffer pH 7.0 (100 mM NaCl, 10 mM TrisCl pH 7.0,1 mM EDTA, 0.5% SDS) with addition of 10 μg Proteinase K and incubated at 50 °C for 45 min with end-to-end shaking at 400 rpm. Formaldehyde cross-links were reversed by incubation at 65 °C with rotation for 4 h. RNA was extracted from these samples using Trizol according to the manufacturer’s protocol (Invitrogen) and treated with DNA-free DNase Treatment & Removal I kit according to the manufacturer’s protocol (Ambion, Austin, TX). The presence of RNA was measured by quantitative RT-PCR using the CFX96 Touch™ Real-Time PCR Detection System (Bio-Rad). Gene-specific PCR primer pairs are listed in Supplementary Table 1. The following antibodies were used for RIP analysis: anti-NF-κB p65 (Santa Cruz), normal mouse IgG (Santa Cruz).

### Chromatin immunoprecipitation assay

Chromatin immunoprecipitation (ChIP) assays were performed as described previously [31]. Briefly, cells were fixed with 1% formaldehyde for 10 min, collected in ice-cold PBS, and resuspended in an SDS lysis buffer. Genomic DNA was then sheared to lengths ranging from 200 to 1000 bp by sonication. One percent of the cell extracts was taken as input, and the rest of the extracts was incubated with either anti-NF-κB p65 (Santa Cruz), anti-H3K4me3 (Cell Signaling), anti-RNA Polymerase 2 (Millipore), or normal mouse IgG (Santa Cruz) overnight at 4 °C, followed by precipitation with protein G-agarose beads. The immunoprecipitates were sequentially washed once with a low-salt buffer, once with a high-salt buffer, once with an LiCl buffer, and twice with a Tris buffer. The DNA–protein complex was eluted, and proteins were then digested with proteinase K for 1 h at 45 °C. The DNA was detected by real-time quantitative PCR analysis. Gene-specific PCR primer pairs are listed in Supplementary Table 1.

### Chromatin isolation by RNA purification

ChIRP analysis was performed as previously reported [31]. Briefly, a pool of tiling oligonucleotide probes with affinity specific to the Nostrill sequence was used and glutaraldehyde cross-linked for chromatin isolation. The sequences for each probe are listed in Supplementary Table 1; probe 1, 3, 5, and 7 are mixed as the probe pool Odd and probe 2, 4, 6, and 8 as the probe pool Even. The DNA sequences of the chromatin isolates were confirmed and quantified by real-time PCR using the same primer sets covering the gene promoter regions of interest as for ChIP analysis. A pool of oligo probes for LacZ were served as controls. The percent input method was used to normalize the ChIRP data.

### Statistical analysis

Data are expressed as mean values and error bars represent standard error of the mean (SEM). Student *T* test with Bonferroni’s correction or one-way ANOVA followed by Tukey-Kramer post hoc tests were performed where appropriate. For determination of significant differences between percents and for multiple comparisons between culture conditions, two-way ANOVA followed by Tukey-Kramer multiple analyses post hoc tests were used. Values of *p* < 0.05 were considered to be significant.

## Results

### Expression of Nostrill in BV2 and primary microglia in response to TLR4-stimulation

**Fig. 1:**
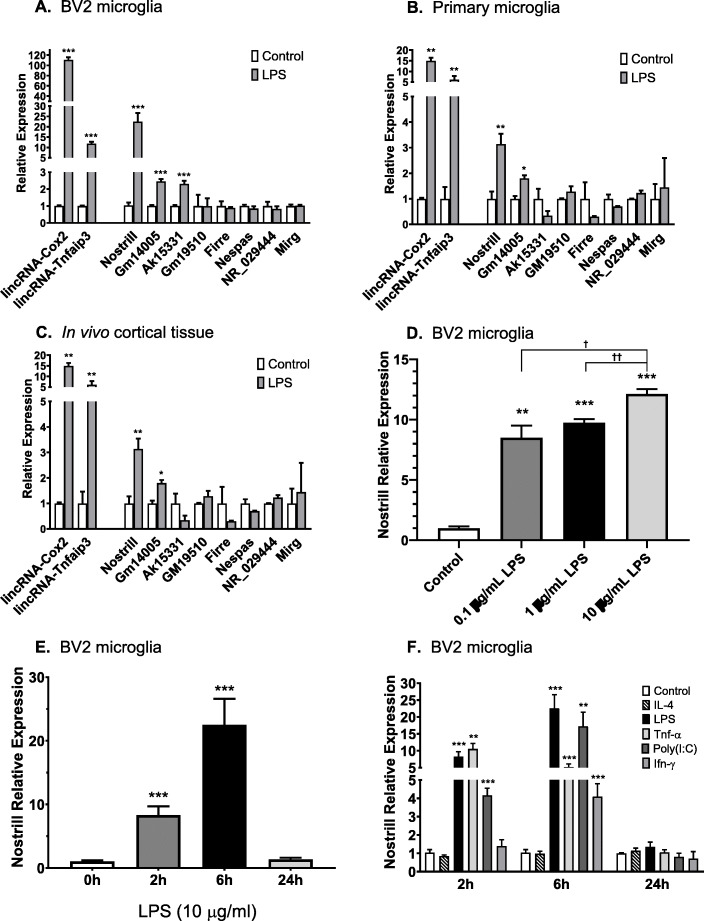
Induction of lncRNA in microglia in response to inflammatory stimuli. **a** Induction of lncRNA in cultured murine microglia (BV2 cells) following stimulation with LPS. BV2 cells were stimulated with 10 μg/ml LPS for 6 h. **b** Induction of lncRNA in primary murine microglia following stimulation with LPS. Primary microglia were stimulated with 10 μg/ml LPS for 6 h. **c** Induction of lncRNA in vivo following stimulation with LPS. C57BL/6 J mice were stimulated with 1 mg/kg LPS or equal volume DPBS for 24 h. RNA was isolated from the cortex region of the brains. **d** Dose response of Nostrill in response to increasing concentrations of LPS. BV2 cells were stimulated with 0.1, 1, or 10 μg/ml LPS for 6 h. **e** Temporal expression of Nostrill in response to LPS. BV2 cells were stimulated with 10 μg/ml LPS for 2, 6, or 24 h. Unstimulated BV2 cells served as controls for each time point. **f** Time-course induction of Nostrill in response to stimulation by inflammatory or anti-inflammatory stimuli. BV2 cells were stimulated with IL-4 (20 ng/ml), LPS (10 μg/ml), TNF-α (20 ng/ml), Poly(I:C) (10 μg/ml), or IFN-γ (20 ng/ml) for 2, 6, or 24 h. Unstimulated BV2 cells served as controls for each time point. Data represent means ± SEM from three independent experiments. Expression levels were validated by real-time quantitative PCR. Gapdh was used as a reference gene for normalization. *p < 0.05, **p < 0.01, and ***p < 0.001 vs control. †*p* < 0.05, and ††*p* < 0.01 between indicated groups

We have previously presented genome-wide RNA transcriptome analysis of mouse microglial BV2 cells stimulated with TLR4 ligand lipopolysaccharide (LPS) using the Agilent SurePrint G3 Mouse Gene Expression Microarray (G4852A, https://www.chem.agilent.com/store/en_US/Prod-G4852A/G4852A) [31]. A total of 5735 lincRNAs such as lincRNA-Cox2 [31] and lincRNA-Tnfaip3 [32] were upregulated in LPS-stimulated BV2 microglia compared to unstimulated controls (ArrayExpress database: E-MTAB-3450) [31]. Here, we confirm the upregulation of lincRNA-Cox2 and lincRNA-Tnfaip3 in response to LPS and demonstrate that three additional lincRNAs were also significantly upregulated in response to LPS stimulation in BV2 cells (Fig. 1a) and in mouse primary microglia (Fig. 1b). Quantitative real-time PCR analyses showed that Nostrill was increased 22.5 ± 4.1-fold, GM14005 was increased 2.45 ± 0.14-fold, and AK15331 was increased 2.30 ± 0.18-fold in LPS-stimulated BV2 cells compared to unstimulated controls (Fig. 1a). LincRNA expression in primary mouse microglia was examined following LPS stimulation. Purity of primary mouse microglial was determined by immunocytochemistry (Supplementary Fig. 1). In primary cultured microglial cells stimulated with LPS, lincRNA-Cox2, and lincRNA-Tnfaip3 levels increased 14.9 ± 1.5-fold and 6.10 ± 1.5-fold, respectively, compared to unstimulated control primary microglia (Fig. 1b). Following LPS stimulation, Nostrill, and GM14005 expression increased 3.14 ± 0.4-fold and 1.80 ± 0.12-fold, respectively, compared to unstimulated control primary microglia (Fig. 1b). LPS stimulation did not significantly upregulate AK15331 expression in primary mouse microglia (Fig. 1b). Several other lincRNAs potentially upregulated in the microarray data set were not confirmed to be upregulated by RT-PCR following LPS-stimulation as compared to unstimulated control BV2 or primary microglia (Fig. 1a-b). This same panel of lincRNAs was evaluated in cortical tissue isolated from an LPS-injection mouse model system at 24 h after LPS-injection (Fig. 1c). RT-PCR analysis of lincRNA expression in the cortical tissue of three LPS-injected mice compared to cortical tissue of three control-injected mice demonstrated that previously characterized lincRNA-Cox2 was significantly upregulated 2.50 ± 0.31-fold (Fig. 1c). Nostrill was also significantly upregulated 3.30 ± 0.84-fold (Fig. 1c). GM14005 and AK15331 were not upregulated or not expressed at detectable levels in the cortical tissues of this in vivo LPS-injected mouse model system (Fig. 1c). While not upregulated in culture cells in response to LPS, NR_029444 was upregulated 4.51 ± 1.24-fold in vivo (Fig. 1c). Since Nostrill was the most highly upregulated lincRNA following LPS stimulation in both BV2 and primary microglial cells and was significantly upregulated in the in vivo LPS-injection mouse model system, Nostrill was chosen for further investigation.

Nostrill upregulation following LPS stimulation increased in a dose-dependent manner up to ~ 12-fold that of unstimulated control levels when BV2 microglia were incubated with LPS at 10 μg/ml (Fig. 1d). Real-time PCR analysis was used to determine the time course of Nostrill expression after LPS exposure. Temporal expression Nostrill in response to LPS stimulation increased to ~ 8-fold above control levels at 2 h of TLR4 stimulation and peaked at 6 h to 22.5 ± 2.08-fold. Nostrill levels returned to baseline by 24 h (Fig. 1e). Nostrill expression in BV2 microglia also increased in response to other known proinflammatory mediators, including tumor necrosis factor (TNF-α), the TLR3 ligand polyinosinic:polycytidylic acid (Poly (I:C)), and Interferon gamma (IFN-γ) (Fig. 1f). Interestingly, Nostrill expression is not influenced by stimulation with the anti-inflammatory cytokine, Interleukin-4 (IL-4) (Fig. 1f).

### Nostrill is a NF-κB responsive gene

**Fig. 2:**
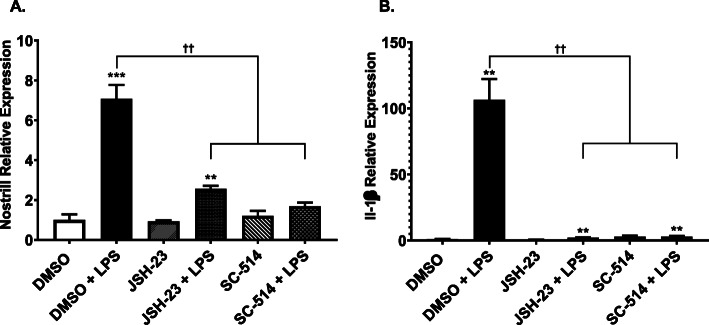
Transcriptional control of Nostrill by the NF-кB signaling pathway in murine microglia following LPS stimulation. **a** Effects of inhibition of the NF-кB signaling pathway on the induction of Nostrill in response to LPS stimulation. BV2 cells were pre-treated with the NF-кB inhibitors SC-514 (100 μM) or JSH-23 (30 μM) 1 h prior to LPS (10 μg/ml) stimulation for 6 h. DMSO was used for stimulation of control cells. **b** Effects of inhibition of NF- кB signaling on the expression of IL-1β, a NF-кB target gene. Data represent means ± SEM from three independent experiments. Expression levels were validated by real-time quantitative PCR. Gapdh was used as a reference gene for normalization. *p < 0.05, **p < 0.01, and ***p < 0.001 vs control. ††*p* < 0.01 between indicated groups

Since NF-κB is a master regulator of proinflammatory responses, we investigated whether blocking NF-κB signaling influences Nostrill expression. Two different NF-κB inhibitors, JSH-23 and SC-514, were used to examine the dependence of Nostrill expression on NF-κB signaling. LPS stimulation increased Nostrill expression 7.08 ± 0.7-fold compared to controls (Fig. 2a). JSH-23 (30 μM) reduced Nostrill expression to 2.56 ± 0.1-fold while SC-514 (100 μM) reduced expression to 1.69 ± 0.2-fold that of controls (Fig. 2a). JSH-23 and SC-514 effectively inhibited IL-1β mRNA expression demonstrating the efficacy of these inhibitors to block NF-κB-mediated gene transcription at the concentrations used for these studies (Fig. 2b).

### Knockdown or overexpression of Nostrill attenuates the upregulation of inflammatory genes triggered by LPS

**Fig. 3:**
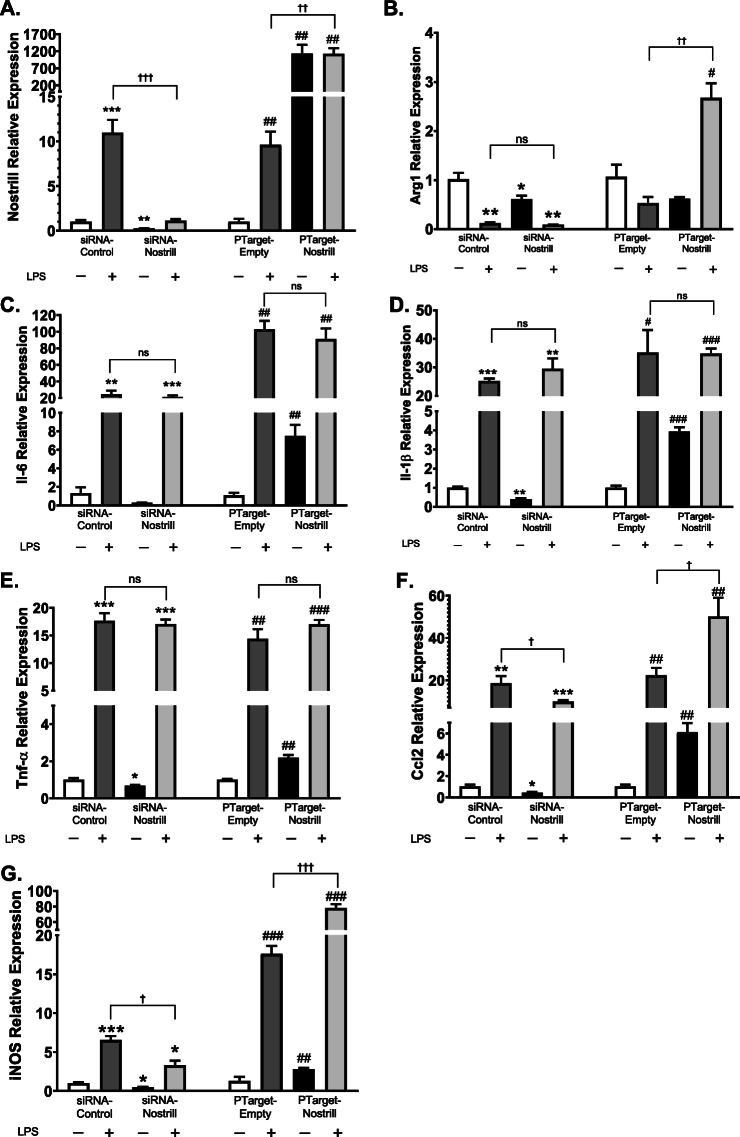
Effect of Nostrill induction on expression of inflammatory genes in microglial cells following LPS stimulation. **a** Validation of knockdown and overexpression of Nostrill in microglial cells. For knockdown, BV2 cells were treated with the designed siRNA to Nostrill for 24 h and subsequently stimulated with LPS (10 μg/ml) for 6 h. A non-specific siRNA sequence was used as the control. For overexpression, BV2 cells were transfected with the PTarget-Nostrill vector for 24 h and then stimulated with LPS (10 μg/ml) for 6 h. BV2 cells treated with the empty PTarget vector were used as the control. Expression levels of selected inflammatory genes, **b** Arg1, **c** IL-6, **d** IL-1β, **e** TNF-α, **f** Ccl2, **g** iNOS, were quantified by using real-time PCR. Gapdh was used as a reference gene for normalization. Data represent means ± SEM from three independent experiments. *p < 0.05, **p < 0.01, and ***p < 0.001 vs control siRNA. #*p* < 0.05, ##*p* < 0.01, and ###*p* < 0.001 vs empty vector. †*p* < 0.05, ††*p* < 0.01, and †††*p* < 0.001 between indicated groups

Since activity of the NF-κB pathway is involved in Nostrill upregulation following TLR4 stimulation, we sought to determine whether knockdown or overexpression of Nostrill affected the expression of NF-κB-responsive genes. Short interfering RNA (siRNA) targeting Nostrill was used to knockdown Nostrill expression. SiRNA-Nostrill significantly reduced basal levels of Nostrill to 0.25 ± 0.1-fold compared to unstimulated control levels (Fig. 3a). Silencing Nostrill in BV2 cells and then stimulating with LPS reduced Nostrill upregulation to that of basal levels seen in unstimulated cells (Fig. 3a). Scrambled siRNA (siRNA-control) did not block LPS-induced upregulation of Nostrill in BV2 cells (Fig. 3a). The overexpression construct (using the PTarget mammalian expression vector) was used to enhance Nostrill expression (PTarget-Nostrill). Real-time PCR showed that BV2 microglia transfected with PTarget-Nostrill increased Nostrill over 1300-fold compared to PTarget control construct (PTarget-empty) that served as the controls (Fig. 3a). Quantitative real-time PCR was used to examine mRNA expression of several downstream target genes of NF-κB signaling when Nostrill was silenced or overexpressed (Fig. 3b-g). Silencing of Nostrill reduced anti-inflammatory cytokine Arginase 1 (Arg1) mRNA levels significantly compared to siRNA-control in the absence of LPS stimulation (Fig. 3b); however, in the Arg1 mRNA levels, there were no significant differences in the presence of LPS stimulation between siRNA-control and siRNA-Nostrill (Fig. 3b). Overexpression of Nostrill without LPS stimulation did not significantly change Arg1 mRNA however with LPS stimulation, Arg1 mRNA levels increased significantly 2.7 ± 0.30-fold compared to PTarget-empty-unstimulated control (*p* < 0.05, Fig. 3b). LPS stimulation of siRNA-control-transfected cells significantly enhanced IL-6, IL-1β, and TNF-α mRNA levels (Fig. 3c-e) but silencing of Nostrill did not significantly affect the increases in IL-6, IL-1β, and TNF-α mRNA (Fig. 3c-e). In the absence of LPS stimulation, silencing of Nostrill in BV2 cells significantly reduced IL-1β and TNF-α mRNA levels but not IL-6 mRNA levels (Fig. 3c-e). Monocyte chemoattractant protein 1 (MCP1/Ccl2) and inducible nitric oxide synthase (iNOS) mRNA levels were significantly reduced in siRNA-Nostrill-transfected BV2 microglia following LPS stimulation when compared to LPS-stimulated siRNA-control (Fig. 3f-g). SiRNA-Nostrill transfection in the absence of LPS stimulation significantly decreased Ccl2 and iNOS (Fig. 3f-g). Overexpression of Nostrill (PTarget-Nostrill) significantly increased IL-6, IL-1β, TNF-α, Ccl2, and iNOS in the absence of LPS stimulation as compared to unstimulated PTarget-empty control cells (Fig. 3c-g). Interestingly, overexpression of Nostrill followed by LPS stimulation significantly increased Ccl2 ~ 2-fold more than in LPS-stimulated PTarget-empty cells (Fig. 3f) and iNOS expression ~4-fold more than in LPS stimulated PTarget-empty cells (Fig. 3g). However, there was not a significant increase in mRNA levels of IL-6, IL-1β, and TNF-α in LPS stimulated PTarget-Nostrill-transfected cells compared to LPS stimulated PTarget-empty-transfected control cells (Fig. 3c-e).

**Fig. 4:**
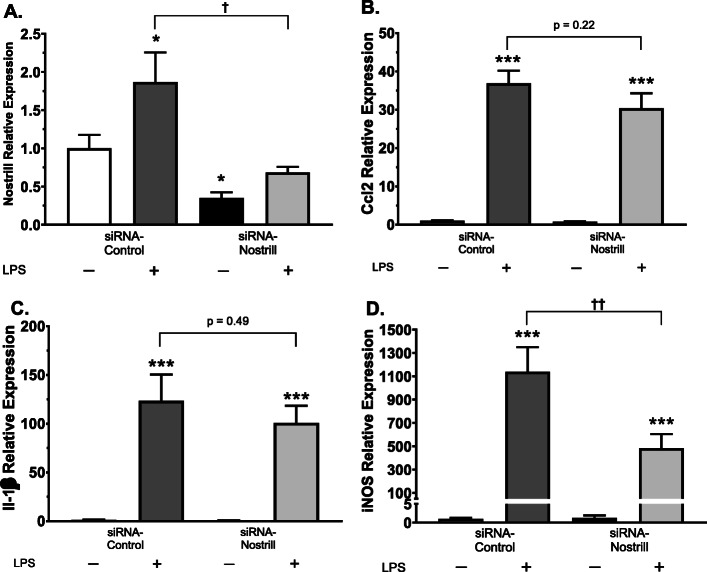
Effect of Nostrill induction on expression of inflammatory genes in primary murine microglia following LPS stimulation. **a** Validation of knockdown of Nostrill in primary murine microglia. For knockdown, primary microglia were treated with the designed siRNA to Nostrill for 24 h and subsequently stimulated with LPS (10 μg/ml) for 6 h. A non-specific siRNA sequence was used as the control. Expression levels of selected inflammatory genes, **b** Ccl2, **c** IL-1β, **d** iNOS, were quantified by using real-time PCR. Gapdh was used as a reference gene for normalization. Data represent means ± SEM from three independent experiments. *p < 0.05, **p < 0.01, and ***p < 0.001 vs control siRNA. †*p* < 0.05, and ††*p* < 0.01 between indicated groups

Nostrill was also silenced in primary microglia (Fig. 4). LPS stimulation of primary microglia transfected with scrambled siRNA-control increased Nostrill 2.2 ± 0.5-fold compared to unstimulated siRNA-control (Fig. 4a). SiRNA-Nostrill transfection of primary microglia significantly reduced Nostrill expression in unstimulated microglia to 0.35 ± 0.07-fold that of siRNA-control unstimulated controls (*p* < 0.05, Fig. 4a). SiRNA -Nostrill significantly reduced Nostrill expression following LPS stimulation to 0.68 ± 0.07-fold (*p* < 0.05, Fig. 4a). SiRNA-Nostrill did not significantly affect Ccl2 (Fig. 4b) or IL-1β (Fig. 4c) mRNA expression in LPS-stimulated primary microglia compared to stimulated siRNA-control microglia but did significantly reduce iNOS mRNA expression (Fig. 4d). Since silencing of Nostrill reduced iNOS mRNA expression in both BV2 (Fig. 3) and primary microglia (Fig. 4), we investigated the relationship between Nostrill and iNOS expression further.

### Loss- or gain-of-function of Nostrill regulates the production of nitric oxide by LPS-stimulated BV2 and primary microglia

**Fig. 5:**
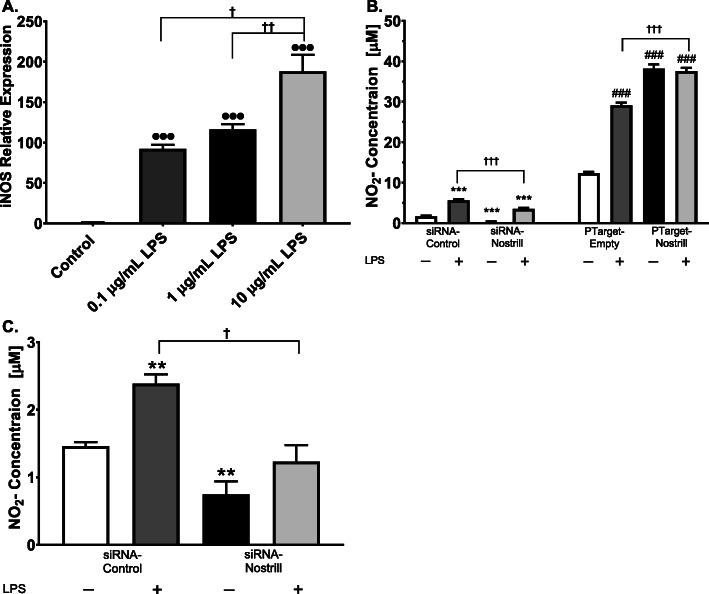
Effect of Nostrill induction on production of nitric oxide in microglia following LPS stimulation. **a** Dose response of iNOS in response to increasing concentrations of LPS. BV2 cells were stimulated with 0.1, 1, or 10 μg/ml LPS for 6 h. Expression levels were validated by real-time quantitative PCR. Gapdh was used as a reference gene for normalization. **b** Production of nitric oxide in BV2 cells following knockdown or overexpression of Nostrill. BV2 cells were transfected with the designed siRNA to Nostrill, a scrambled siRNA-control, the PTarget-Nostrill expression vector, or the empty PTarget vector for 24 h. Cells were stimulated with LPS (10 μg/ml) for 6 h. Media was collected and the Griess assay was performed. **c** Production of nitric oxide in primary murine microglia following knockdown of Nostrill. Primary murine microglia were transfected with the designed siRNA targeting Nostrill or a control scrambled siRNA for 24 h, then stimulated with LPS (10 μg/ml) for 6 h. Media was collected and the Griess assay was performed. Data represent means ± SEM from three independent experiments. ●●●*p* < 0.001 vs control. *p < 0.05, **p < 0.01, and ***p < 0.001 vs control siRNA. #*p* < 0.05, ##*p* < 0.01, and ###*p* < 0.001 vs empty vector. †*p* < 0.05, ††*p* < 0.01, and †††*p* < 0.001 between indicated groups

In BV2 microglia, *iNOS* gene transcription increased significantly with doses of LPS from 0-10 μg/ml (Fig. 5a). Real-time PCR analyses showed that at 10 μg/ml, iNOS mRNA levels reached 188.2 ± 20.3-fold that of control, unstimulated levels (Fig. 5a). The concentration of 10 μg/ml LPS was used to stimulate siRNA-control- and siRNA-Nostrill-transfected BV2 microglia as well as PTarget-empty and PTarget-Nostrill-transfected microglia to assess NO_2_ production using Griess assays (Fig. 5b). In the absence of LPS stimulation, silencing of Nostrill significantly reduced NO_2_ production in BV2 microglia compared to untreated, control transfected cells (Fig. 5b). Treatment with LPS significantly increased NO_2_ production detected in control transfected cells as compared to untreated, control transfected cells (Fig. 5b). Silencing of Nostrill significantly decreased NO_2_ production following LPS treatment as compared to LPS treated, siRNA-control transfected cells (*p* < 0.001, Fig. 5b). The level of NO_2_ production following silencing of Nostrill and LPS treatment was significantly different from untreated siRNA-control and siRNA-Nostrill-transfected cells that were not treated with LPS (Fig. 5b). Overexpression of Nostrill (PTarget-Nostrill) in BV2 microglia significantly increased NO_2_ production as compared to untreated and LPS-treated PTarget-empty-transfected control cells (*p* < 0.001, Fig. 5b). LPS treatment of PTarget-Nostrill cells did not significantly increase NO_2_ production in BV2 microglial as compared to untreated PTarget-Nostrill-transfected cells (Fig. 5b). In primary microglial cells, silencing of Nostrill significantly decreased NO_2_ production in unstimulated siRNA-Nostrill-transfected primary microglia as compared to unstimulated control cells (Fig. 5c). Following LPS stimulation, silencing of Nostrill significantly reduced NO_2_ production in siRNA-Nostrill-transfected cells as compared to stimulated siRNA-control-transfected cells (Fig. 5c).

### Nostrill promotes iNOS transcription through chromatin modifications associated with the p65 protein of the NF-κB subunits

**Fig. 6:**
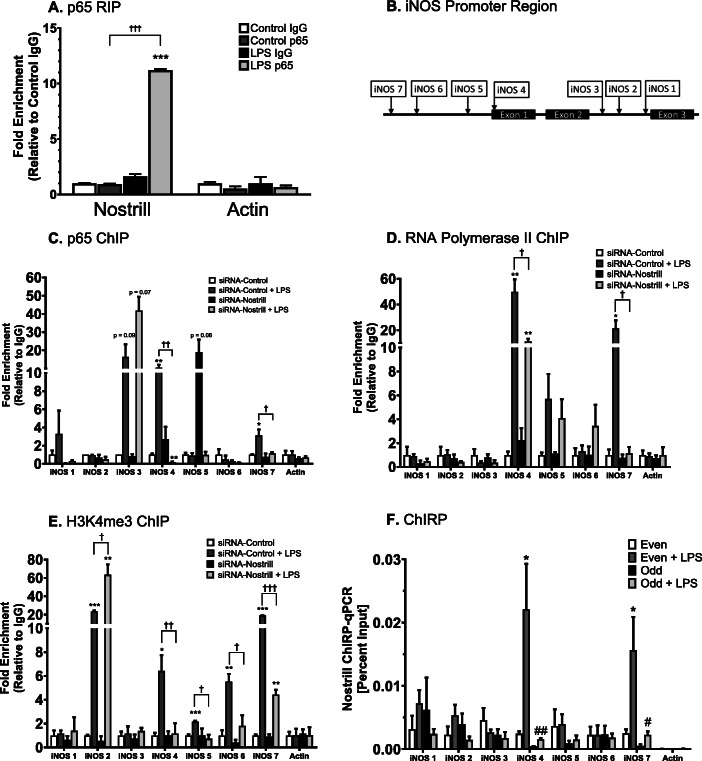
Interaction of Nostrill and NF-кB p65 and impact of Nostrill induction on the transcriptional control of iNOS. **a** Physical interaction of Nostrill with NF-кB p65 in BV2 cells. BV2 cells were exposed to LPS (10 μg/ml) for 6 h, followed by RNA immunoprecipitation (RIP) analysis using anti-p65 or normal IgG. Presence of Nostrill, but not the control Actin in the immunoprecipitates from stimulated cells were detected by real-time PCR. ***p < 0.001 vs control IgG. †††*p* < 0.001 between indicated groups. **b** Diagram of primer sets of iNOS promoter region. **c** Impact of Nostrill on recruitment of NF-кB p65 to the iNOS gene locus in BV2 cells following LPS stimulation. BV2 cells were transfected with the Nostrill siRNA or control scrambled siRNA for 24 h, then stimulated with LPS (10 μg/ml) for 6 h, followed by ChIP analysis using anti-p65 and the PCR primer sets as designed. *p < 0.05, **p < 0.01, and ***p < 0.001 vs control siRNA. †*p* < 0.05 and ††*p* < 0.01 between indicated groups. **d** Impact of Nostrill on recruitment of RNA polymerase II to the iNOS gene locus in BV2 cells following LPS stimulation. BV2 cells were transfected with the Nostrill siRNA or control scrambled siRNA for 24 h, then stimulated with LPS (10 μg/ml) for 6 h, followed by ChIP analysis using anti-Pol2 and the PCR primer sets as designed. *p < 0.05, **p < 0.01, and ***p < 0.001 vs control siRNA. †*p* < 0.05 between indicated groups. **e** Impact of Nostrill on activating histone modifications at the iNOS gene locus in BV2 cells following LPS stimulation. BV2 cells were transfected with the Nostrill siRNA or control scrambled siRNA for 24 h, then stimulated with LPS (10 μg/ml) for 6 h, followed by ChIP analysis using anti-H3K4me3 and the PCR primer sets as designed. *p < 0.05, **p < 0.01, and ***p < 0.001 vs control siRNA. †*p* < 0.05, ††*p* < 0.01, and †††*p* < 0.001 between indicated groups. **f** Increased recruitment of Nostrill to the iNOS gene locus in BV2 cells following LPS stimulation. BV2 cells were stimulated with LPS (10 μg/ml) for 6 h, followed by ChIRP analysis using two pools of probes specific to Nostrill and the PCR primer sets as designed. Probes for LacZ served as a negative control. *p < 0.05 vs even probe pool. #*p* < 0.05, ##*p* < 0.01 vs odd probe pool. Data represent means ± SEM from three independent experiments

Given that Nostrill expression is associated with NF-κB signaling, upregulation of iNOS mRNA, and production of nitric oxide, we sought to determine whether Nostrill can directly interact with NF-κB subunit proteins at iNOS promoter regions. Formaldehyde crosslinking RNA immunoprecipitation (RIP) analysis was performed on LPS stimulated and unstimulated BV2 microglia (Fig. 6a). Immunoprecipitation of NF-κB p65 demonstrated an 11.2 ± 0.1-fold of Nostrill enrichment in LPS stimulated cells as compared to control-untreated cells (Fig. 6a). Immunoprecipitation using a non-specific IgG antibody did not demonstrate enrichment of Nostrill in LPS stimulated or control cells (Fig. 6a). No significant interaction between actin and NF-κB p65 was observed in LPS stimulated or control cells (Fig. 6a). We used chromatin immunoprecipitation (ChIP) assays and silencing of Nostrill expression to evaluate Nostrill involvement in docking NF-κB p65 at *iNOS* promoter region. Primer sets positioned at several different locations within the *iNOS* promoter region (iNOS 1-7) were used to determine whether silencing of Nostrill influenced the association of p65 to *iNOS* promoter region (Fig. 6b). Enrichment of p65 at *iNOS* promoter regions 4 and 7 was significant in siRNA-control-transfected BV2 cells following LPS stimulation compared to controls (Fig. 6c). Enrichment of NF-κB p65 at iNOS promoter region 3 in LPS-stimulated siRNA-control was not significant (*p* = 0.09). Enrichment of NF-κB p65 at iNOS promoter region 3 was also not significant in the LPS-stimulated siRNA-Nostrill condition (*p* = 0.07, Fig. 6c). Silencing of Nostrill did not significantly reduce amplification of *iNOS* transcriptional region by primer sets 4 and 7 following cross-linking to p65 in unstimulated BV2 cells (Fig. 6c). Importantly, silencing of Nostrill in LPS stimulated BV2 cells significantly reduced enrichment of the *iNOS* transcriptional region amplified by primer sets 4 and 7 to 0.19 ± 0.05-fold and 1.14 ± 0.15-fold, respectively (Fig. 6c). In unstimulated and LPS stimulated BV2 cells, siRNA-control and siRNA-Nostrill treatments did not show enrichment for association with genomic actin transcription site (Fig. 6c). ChIP analyses also showed that following LPS stimulation, crosslinking of RNA Polymerase II (Pol II) to the chromatin of siRNA-control-transfected BV2 cells showed significant enrichment of *iNOS* transcriptional region amplified by primer sets 4 and 7 to 49.9 ± 9.6-fold and 21.4 ± 6.2-fold, respectively (Fig. 6d). Silencing of Nostrill significantly reduced enrichment of *iNOS* transcriptional region by primer sets 4 and 7 to 11.36 ± 2.0-fold and 1.14 ± 0.53-fold, respectively (Fig. 6d).

Modification of histone proteins such as H3 is necessary for gene transcription. Commonly, the tri-methylation of H3K4 (H3K4me3) is associated with and is necessary for enhanced transcription of nearby genes. ChIP analysis using H3K4me3 antibodies demonstrated an enhanced association of H3K4me3 with *iNOS* transcriptional region amplified by primer sets 4 and 7 as well as other locations within the *iNOS* transcriptional region by primer sets 2, 5, and 6 (Fig. 6e). Primer sets 5 and 6 show enrichment following LPS stimulation in siRNA-control-transfected BV2 cells and inhibition of enrichment in siRNA-Nostrill transfected LPS-stimulated BV2 cells (Fig. 6e). Primer set iNOS 2 demonstrated enrichment following LPS stimulation in siRNA-control and siRNA-Nostrill-transfected BV2 cells (Fig. 6e).

To further examine whether Nostrill is directly associated with the chromatin modifications necessary for iNOS transcription, chromatin isolation by RNA purification (ChIRP) was performed (Fig. 6f). For increased sensitivity and specificity, probes to Nostrill were split between even and odd pools to test whether Nostrill was directly recruited to the *iNOS* promoter region. Following pull-down of Nostrill biotinylated probes, quantitative PCR demonstrated increased interactions of Nostrill and iNOS promoter region at the same sets 4 and 7 locations as for p65 docking and Pol II recruitment and H3K4me3 enrichment in BV2 cells following LPS stimulation (Fig. 6f). Increased interactions were detected in both even and odd probe pools (Fig. 6f). Quantitative PCR using genomic actin primers did not show interactions between Nostrill and serves as negative control (Fig. 6f).

### Effect of Nostrill expression on the neurotoxicity of LPS-stimulated microglia

**Fig. 7:**
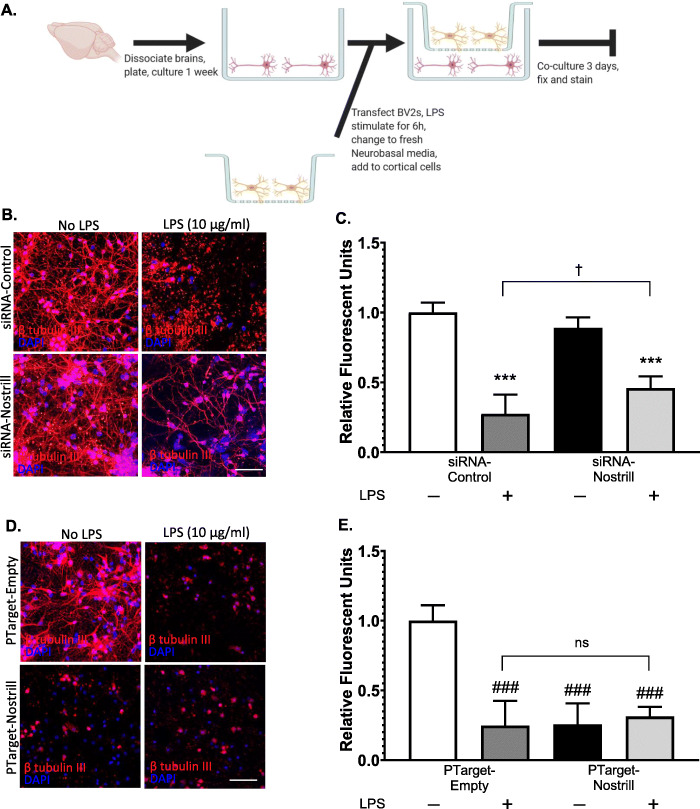
Effect of Nostrill induction on microglial-mediated neurotoxicity. **a** Illustration of co-culture experiments. Briefly, brains were dissected, cortices were dissociated, and cortical cells were cultured for 1 week. BV2s were plated on transwells, transfected, and stimulated with LPS (10 μg/ml) for 6 h. Media was then exchanged for fresh Neurobasal media, and the transwells containing stimulated BV2s were transferred to co-culture with the cortical cells for 3 days. Cortical cells were subsequently fixed and stained. Illustration created using BioRender. **b** Impact of Nostrill knockdown on microglial-mediated neurotoxicity. Cortical cells were stained with β-tubulin III and DAPI. Scale bar = 50 μm. Relative fluorescent units were quantified in (**c**). ***p < 0.001 vs siRNA-control. †*p* < 0.05 between indicated groups. **d** Impact of Nostrill overexpression on microglial-mediated neurotoxicity. Cortical cells were stained with neuron-specific, β-tubulin III, and DAPI. Scale bar = 50 μm. Relative fluorescent units were quantified in (**e**). ###*p* < 0.001 vs empty vector. Data represent means ± SEM from three independent experiments

To investigate whether silencing Nostrill reduced microglial neurotoxicity, we designed co-culture experiments where BV2 microglia were transfected with silencing or overexpression constructs, stimulated with LPS (10 μg/ml), washed to remove LPS, and then co-cultured with cortical neurons for 3 days in vitro (Fig. 7a). At the end of co-culturing, neurons were fixed and immunostained for β tubulin III, a neuronal-specific cytoskeletal associated protein that is localized to neuronal processes. Neuronal cell bodies were detected by DAPI nuclear staining (Fig. 7a, b). Extensive neuronal differentiation and neuronal process outgrowth following co-culture with siRNA-control- and siRNA-Nostrill-transfected microglia were observed (Fig. 7b). LPS stimulation of siRNA-control-transfected cells resulted in significant loss of immunoreactivity to β tubulin III seen in healthy neuronal processes (Fig. 7b). Co-culture of neurons with LPS stimulated BV2 cells following siRNA-Nostrill transfection noticeably reduced the dramatic loss of β tubulin III expression and loss of neuronal processes seen in LPS stimulated, siRNA-control-transfected BV2 co-cultures (Fig. 7b). Relative fluorescence of β tubulin III immunoreactivity was quantified and normalized to the unstimulated siRNA-control transfection condition (Fig. 7c). Silencing of Nostrill in LPS-stimulated BV2 microglia significantly improved β tubulin III immunoreactivity by ~ 20% fold (*p* < 0.05, Fig. 7c). Overexpression of Nostrill in microglia resulted in co-cultured neurons with little β tubulin III immunoreactivity (Fig. 7d). LPS-stimulation of BV2 microglia overexpressing Nostrill also resulted in co-culture conditions that did not support neuronal expression of β tubulin III (Fig. 7d). Unstimulated BV2 cells transfected with PTarget control did not affect β tubulin III expression and neurite outgrowth (Fig. 7d). Quantification of β tubulin III immunoreactivity in cortical neurons co-cultured with LPS-stimulated PTarget-transfected BV2s was significantly decreased by ~ 75% that of unstimulated PTarget-transfected controls (Fig. 7e, *p* < 0.001). An ~ 74% reduction in β tubulin III immunoreactivity was observed in cortical neurons co-cultured with unstimulated BV2 cells overexpressing Nostrill (PTarget-Nostrill) as compared to unstimulated PTarget-transfected controls (Fig. 7e). LPS stimulation of BV2 cells overexpressing Nostrill did not demonstrate a significant synergistic effect on β tubulin III immunoreactivity as compared to LPS-stimulated PTarget-empty controls (Fig. 7e, *p* > 0.05).

**Fig. 8:**
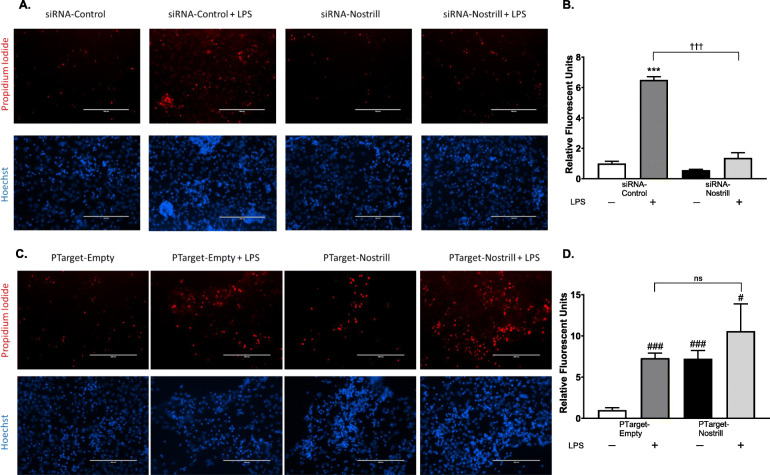
Impact of Nostrill induction on microglial-mediated neurotoxicity. **a** Impact of Nostrill knockdown on microglial-mediated neurotoxicity. BV2 cells were transfected with siRNA for 24 h, stimulated with LPS (10 μg/ml) for 6 h, then media was exchanged for fresh media. BV2 cells conditioned media for 72 h, before that media was placed on cortical cells. Cortical cells were stained with propidium iodide and Hoechst. Scale bar = 200 μm. Relative fluorescent units were quantified in (**b**). ***p < 0.001 vs siRNA- control. †††*p* < 0.001 between indicated groups. **c** Impact of Nostrill overexpression on microglial-mediated neurotoxicity. Cortical cells were stained with propidium iodide and Hoechst. Scale bar = 200 μm. Relative fluorescent units were quantified in (**d**). #*p* < 0.05, ###*p* < 0.001 vs empty vector. Data represent means ± SEM from three independent experiments

Propidium iodide (PI) staining is commonly used as a reliable indicator of cell death in vitro as it is excluded from living cells and taken up by dying or dead cells [40]. Using the co-culture experimental design (Fig. 7a), neurons were stained with propidium iodide after co-culture with LPS-stimulated BV2 microglia following silencing and overexpression of Nostrill. Unstimulated and control transfected BV2 cells (siRNA-control and PTarget-empty) served as controls (Fig. 8). LPS stimulation of siRNA-Control-transfected microglia significantly increased PI staining to 6.5 ± 0.20-fold that of unstimulated controls (Fig. 8a, b). Silencing of Nostrill in unstimulated controls (siRNA-Nostrill) did not significantly reduce PI uptake by neurons as compared to unstimulated siRNA-control conditions but significantly reduced the increased PI uptake following LPS-stimulation (siRNA-Nostrill + LPS) to near control levels at 1.37 ± 0.33-fold (Fig. 8a-b). Overexpression of Nostrill in unstimulated BV2 cells resulted in increased PI uptake in co-cultured neurons to 7.22 ± 0.99-fold that of neurons co-cultured with unstimulated PTarget-empty control BV2 cells (Fig. 8c-d). This increase in PI uptake in neurons was similar to neurons co-cultured with LPS-stimulated PTarget-empty control BV2 cells (Fig. 8c-d). Co-culture of neurons with BV2 cells overexpressing Nostrill and then stimulated with LPS significantly increased PI uptake in neurons to 10.58 ± 3.28-fold that of control co-culture conditions (Fig. 8c-d) but were not significantly different from PI uptake by neurons in LPS-stimulated PTarget-empty or unstimulated PTarget-Nostrill co-culture conditions (Fig. 8d).

## Discussion

Microglial proinflammatory states elicited by systemic immune responses to bacterial or viral pathogens are associated with a variety of neurodegenerative and autoimmune diseases [41–45]. In order to determine whether inhibition of specific molecular mechanisms regulating microglial-mediated neuroinflammation will be effective for prevention or treatment of these diseases, it is necessary to increase our understanding of the molecular processes regulating microglial pro-inflammatory states.

LncRNAs are now known to be important for regulating gene transcription associated with many biological processes [46] including inflammation [25, 47]. How lncRNAs contribute to neuroinflammatory processes of microglial cells remains poorly understood. LincRNAs have diverse functions [48] including regulation of gene expression [49, 50], epigenetic modification [51], pluripotency [52, 53], and proinflammatory responses [54]. We previously published that several lincRNAs are up- or downregulated in LPS-stimulated macrophages and microglia [31]. Here, we report that Nostrill is significantly upregulated in a dose- and time-dependent manner following LPS stimulation in BV2 microglial cell lines and primary microglial cells. Twenty-four hours after LPS injection, Nostrill is also significantly upregulated in cortical brain tissue of LPS-injected mice. The amount of Nostrill upregulation induced by LPS stimulation in BV2 cell lines, primary microglia, and cortical brain tissue differs. These differences may be due to variations in stimulation protocols or to unique mechanisms regulating lincRNA transcription in cell lines, primary cells, and in vivo. Similar results are seen with lincRNA-Cox2 and lincRNA-Tnfaip3 following LPS stimulation (Fig. 1) suggesting that these results are not specific to Nostrill and that future in vivo mechanistic studies are important. Interestingly, Nostrill expression is significantly upregulated in BV2 microglia within 6 h in response to LPS as well as Tnf-α, Poly (I:C), and Ifn-γ but not by IL-4, an anti-inflammatory activator of microglia [55]. These data suggest that Nostrill is important during the beginning stages of microglial activation to a pro-inflammatory state and not an anti-inflammatory state. A handful of lncRNAs such as lincRNA-Cox2 [31, 37], lincRNA p21 [36] lncRNA H19 [56], lncRNA HOTAIR [57, 58], lncRNA MALAT1 [59, 60], and lncRNA TUG1 [61, 62] have also been shown to participate in driving pro-inflammatory polarization of microglia. Recently, lincRNA-Cox2 has also been shown to control cell cycle gene expression and that silencing of lincRNA-Cox2 reduces LPS-induced microglial proliferation [63].

Interestingly, the expression of several of these lncRNAs, such as lincRNA-Cox2 is regulated by the pro-inflammatory transcription factor NF-κB [31, 38, 64]. NF-κB-mediated transcription of lincRNAs likely coincides with the new protein synthesis driven by early response genes [65] and may allow for lincRNA and protein interactions necessary for secondary and late gene transcription. Similar to lincRNA-Cox2, one of the major findings of this study is that LPS-induced upregulation of Nostrill appears to be dependent upon NF-κB signaling since two inhibitors of the NF-κB signaling pathway (SC-514, an IKK-2 inhibitor, and JSH-23, an NF-κB p65 inhibitor) significantly attenuated Nostrill expression in LPS-stimulated BV2 cells (Fig. 2). Silencing of Nostrill in LPS-stimulated BV2 and primary microglia reduced *iNOS* gene transcription (Figs. 3, 4, and 5) and nitric oxide production (Fig. 6). Overexpression of Nostrill in BV2 cells increased inducible NOS (iNOS) mRNA and nitric oxide production (Fig. 6) suggesting that Nostrill acts to drive *iNOS* gene transcription and NO synthesis in LPS-stimulated microglia. Since *iNOS* is a secondary response gene, the regulatory mechanisms of *iNOS* gene transcription may require protein synthesis and chromatin remodeling [66]. RIP analyses showed Nostrill may regulate *iNOS* gene transcription by interacting with NF-κB p65 and then associating with regions within iNOS promoter sites as indicated by ChIP. Silencing of Nostrill revealed further that the assembly of RNA polymerase II and modified histone H3K4me3 at *iNOS* promoter region following LPS stimulation was influenced by Nostrill. Recruitment of Nostrill to the gene locus of the secondary response gene *iNOS* was further confirmed by ChIRP. Interestingly, Nostrill was recently identified as a chromatin architectural protein (CAP) associated lincRNA but Nostrill’s functional role was not studied [67]. As one of two recently identified CAP-associated lincRNAs, Nostrill could readily act as a scaffold in long-range chromatin and protein interactions to assist with *iNOS* transcription [67]. Several reports have speculated that lincRNAs may function as scaffold molecules to affect gene expression [25, 48, 68]. LncRNAs may function as scaffold molecules because they are able to interact with RNA-binding proteins such as polycomb repressive complex 1 (PRC1) or MyBBP1A [24, 69, 70]. Specifically, previous work has shown that lincRNA-Cox2 directly interacts with MyBBP1A and may be necessary for MyBBP1A assembly into the SWI/SNF complex [31]. Studies using lincRNA-Cox2-deficient mice and in vitro methods demonstrate that lincRNA-Cox2 has a trans regulatory role controlling the expression of several immune responsive genes [31, 37, 68, 71]. These data suggest Nostrill may also function to scaffold the transcriptionally active p65 protein of NF-κB, H3K4me3, and RNA polymerase II at *iNOS* promoter region. The assembly of other RNA-binding proteins with Nostrill is undetermined and is under investigation. Since Nostrill, lincRNA-Cox2, and lincRNA-Tnfaip3 are all upregulated through activation of NF-κB signaling and display regulatory effects on the transcription of inflammatory genes, it is possible that they may function coordinately to provide fine-tuned regulation of inflammatory responses in microglia. Future studies investigating the coordinated activity of lincRNAs would provide novel information about NF-κB-regulated inflammatory processes.

Proinflammatory activation of microglia causing the overproduction and/or sustained production of nitric oxide (NO) contributes to neurotoxicity [58, 72]. Mechanisms of neurotoxicity involving lincRNA function in microglia may underly the development and persistence of neurodegenerative and autoimmune disease processes [6, 17, 25, 41, 54]. For example, the antimicrobial immune response of microglia to release NO in response to bacterial infection is known to contribute to the destruction of myelin and death of CNS neurons during proinflammatory phases of multiple sclerosis [13, 41, 73]. Targeted inhibition of proinflammatory pathways in microglia may reduce neurotoxicity and help mitigate or treat such neuroinflammatory disorders [6]. Studies have shown that siRNA delivery to the CNS can exacerbate or reduce aspects of lincRNA-regulated microglial proinflammatory responses in vivo [35, 63, 74, 75] indicating their utility when the lincRNA function is fully understood. Our proof-of-concept, in vitro, co-culture experiments showed that silencing of Nostrill in microglia inhibits LPS-stimulated neurotoxicity while overexpression of Nostrill leads to neurotoxicity (Figs. 7 and 8). Both immunocytochemistry and PI-uptake data in this in vitro system provide support for the hypothesis that blocking proinflammatory, lincRNA-mediated gene transcription can reduce neurotoxicity. Thes*e* in vitro studies are the first step to investigating the neurobiological relevance of targeting Nostrill in microglia following activation by pathogenic signals such as bacterial LPS. Future studies are designed to confirm the function of Nostrill and the potential therapeutic effects of Nostrill silencing using in vivo model systems. Such studies will further expand our understanding of the utility of RNA drug targets for neurodegenerative and autoimmune diseases.

## Supplementary Information

**Additional file 1: Supplementary Fig. 1**. Purity of primary mouse microglial by immunocytochemistry.

**Additional file 2: Supplementary Table 1**. Primers for RT-PCR.

## Data Availability

The datasets used and/or analyzed during the current study are available from the corresponding author on reasonable request.

## Abbreviations

ChIP: Chromatin IP
CHIRP: Chromatin isolation by RNA purification
IP: Immunoprecipitation
P: Postnatal
RT-PCR: Reverse transcriptase-polymerase chain reaction
IFN-γ: Interferon-gamma
TNFα: Tumor necrosis factor alpha
NF-κB: Nuclear factor kappa-light-chain-enhancer of activated B cells
GFAP: Glial fibrillary acidic protein
SEM: Standard error of the mean
O.D.: Optical density
RFU: Relative immunofluorescence units
DAPI: 4′,6-diamino-2-phenylindole
DIV: Days in vitro
DMEM: Dulbecco’s Modified Eagles Media
FBS: Fetal bovine serum
DPBS: Dulbecco’s phosphate-buffered saline
Gapdh: Glyceraldehyde 3-phosphate dehydrogenase
ΔΔCt: Delta-delta cycle threshold
RIP: RNA immunoprecipitation
siRNA: Small interfering RNA
lincRNA: Long intergenic non-coding RNA
lncRNA: Long non-coding RNA
