# Supplementary figures and images for: A novel long intergenic non-coding RNA, Nostrill, regulates iNOS gene transcription and neurotoxicity in microglia
Source: J Neuroinflammation. 2021 Jan 6;18:16. doi: 10.1186/s12974-020-02051-5 (PMC7789650; doi:10.1186/s12974-020-02051-5)

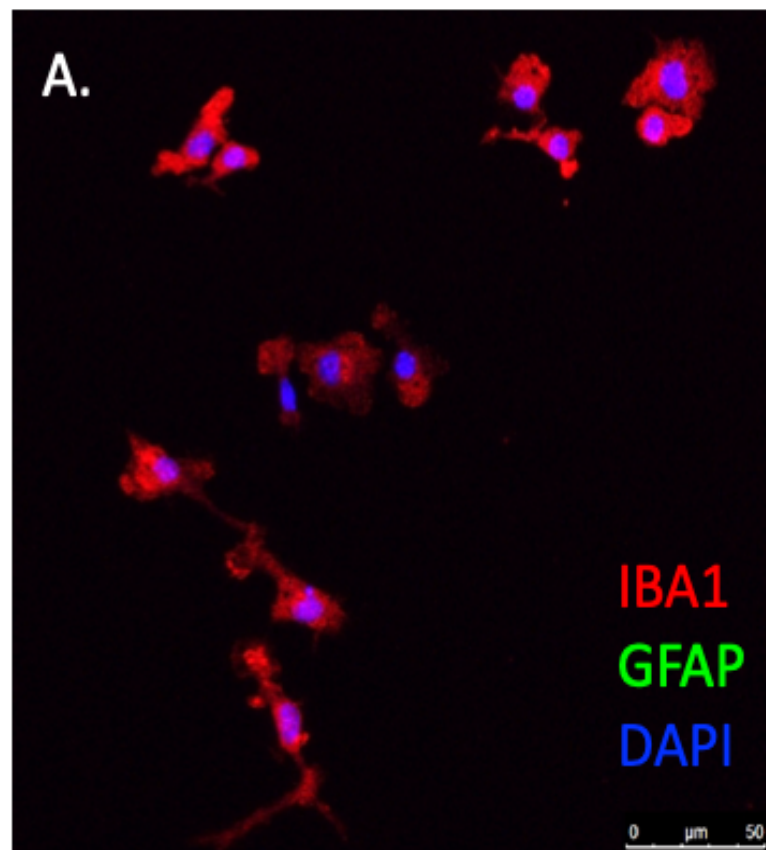

B.

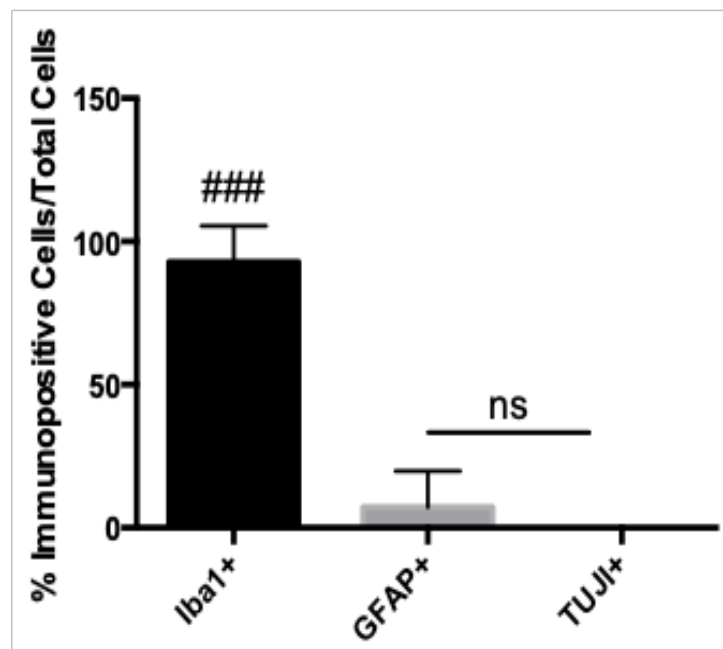

Supplement: Supplementary file 1 — Additional file 1: Supplementary Fig. 1. Purity of primary mouse microglial by immunocytochemistry. [file 12974_2020_2051_MOESM1_ESM.pdf]
